# 

**Published:** 1891-05-23

**Authors:** 


PRICE ONE PENNY.

No. 243, Vol. X.— May 23rd, 1891.

[Registered at the General Post Office as a Newspaper for transmission in the United Kingdom and Abroad.]

WITH EXTRA NURSING SUPPLEMENT.

Per Annum, 6s. 6d.; post free.

Monthly Parts, 6d.; post free 8d.

Ditto per Annum, 8s., post free.

### THE HOSPITAL

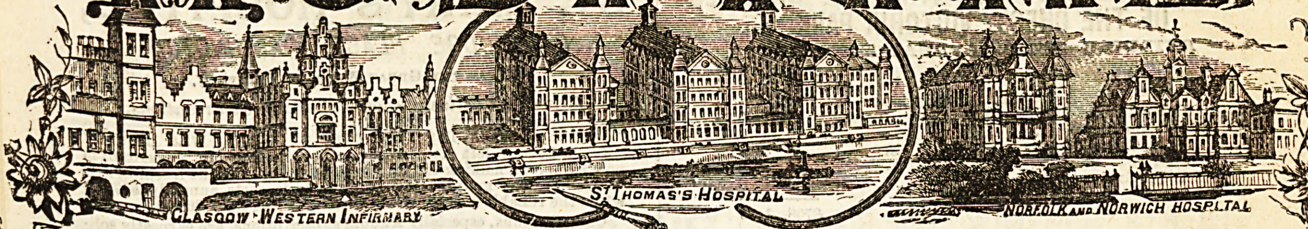

A WEEKLY JOURNAL OF

### Science, Medicine, Nursing and Philanthropy.

SATURDAY, MAY 23rd, 1891.

**Noodles and Newspapers** - - - - 85

**Passing Topics.**—Putting the House in Order - - 86

**The Doctor's Armchair.**—Racial Science and Modern Politics - - - - 87

**The History and Capabilities of Herbal Simples.** By W. T. Fernie, M.D.—XXXI.—The Elder - - 88

**Everybody's Page** - - - - 89

**Notes and News** - - - - 90

**General Charities—Scraps and Gleanings** - - 91

**Annotations.**—A Jewish Lady Centenarian—Weather Science at the House of Commons—The Doctor's Pound of Tea - - - - 92

**The Practitioner's Mirror and Retrospect:—**

Medicine.—Eczema, 93; Diabetic Coma - - - 94

Therapeutics.—Gelsemium Sempervirens - - - 94

New Drugs, Appliances, and Things Medical.—Scrubb's Cloudy Household Ammonia.—Æsculap Water 95

**Books Received** - - - - 95

**Editor's Letter Box.**—The Dulness of Domestic Service - 95

**Neuritis of the Peripheral Nerves.**—II. By J. Michell Clarke, M.A., M.B., M.R.C.P - - - 96

**The Student of Medicine.**—Appointments—Students' Bookshelf—Scholarships and Prizes — Notes from the Schools—Pass Lists—Athletics.

### EXTRA SUPPLEMENT—THE NURSING MIRROR.

**En Passant.**—Lincoln Institution for Nurses—Salford Workhouse—A Storm in a Teacup—Short Items—The Nurses' Co-operation—Workhouse Infirmary Nursing Association - - - xliii

**Lectures on Surgical Ward Work and Nursing.**—By Alex. Miles, M.B. Edin., C.M., F.R.C.S.E.—Lecture XXV.—The Long Splint - - - xliv

**Death in Our Ranks** - - - - xliv

**A First Day** - - - - xlv

**For Reading to the Sick.**—Spring Time - - - xlv

**Notes from Australia** - - - - xlvi

**Nurse Training in the Seventies.**—By E. D. (*cont.*) - xlvi

**Everybody's Opinion.**—Male Nurses - - - xlvii

**The Royal British Nurses' Association.**—Refusal of Licence by the Board of Trade - - - xlvii

**Presentation** - - - - xlvii

**Notes and Queries** - - - - xlvii

**Off Duty.**—Human Flowers - - - x viii

**Appointments** - - - - xlviii

**Amusements and Relaxation** - - - xlviii

